# Biosafety Level-4 Laboratories in Europe: Opportunities for Public Health, Diagnostics, and Research

**DOI:** 10.1371/journal.ppat.1003105

**Published:** 2013-01-17

**Authors:** Carla Nisii, Concetta Castilletti, Hervé Raoul, Roger Hewson, David Brown, Robin Gopal, Markus Eickmann, Stephan Gunther, Ali Mirazimi, Tuija Koivula, Heinz Feldmann, Antonino Di Caro, Maria R. Capobianchi, Giuseppe Ippolito

**Affiliations:** 1 “L. Spallanzani” National Institute for Infectious Diseases, Rome, Italy; 2 French National Institute for Health and Medical Research, Lyon, France; 3 Health Protection Agency, Salisbury, United Kingdom; 4 Health Protection Agency, London, United Kingdom; 5 University of Marburg, Marburg, Germany; 6 Bernhard Nocht Institute of Tropical Medicine, Hamburg, Germany; 7 Swedish Institute for Communicable Disease Control, Solna, Sweden; 8 Division of Intramural Research (DIR), National Institute of Allergy and Infectious Diseases (NIAID), National Institutes of Health (NIH) Rocky Mountain Laboratories, Hamilton, Montana, United States of America; The Fox Chase Cancer Center, United States of America

## How Is Europe Dealing with the Threat of Highly Infectious Pathogens?

Emerging infectious diseases (EIDs) and highly infectious diseases (HIDs) have received much attention in recent years, when a number of networks and projects were funded by the European Community [1]: ENIVD (European Network for the Diagnosis of Imported Viral Diseases), EUNID and EURONHID (European Network of Infectious Disease Physicians), ETHREAT (European Training for Health Professionals on Rapid Responses to Health Threats), ETIDE (European Training for Infectious Disease Emergencies), RIVIGENE (genomic inventory, forensic markers, and assessment of potential therapeutic and vaccine targets for viruses relevant in biological crime and terrorism), and EURONET-P4 and ENP4-Lab (European Network of Biosafety-Level-4 Laboratories). All of the above are examples of initiatives that resulted in sharing of knowledge, practices, and materials [2]–[6]. To address some of the open issues in the area of Biosafety Level-4 (BSL-4) facilities, a 2-day conference was held in Rome on September 9–10, 2010, organized by the “L. Spallanzani” National Institute for Infectious Diseases and co-funded by the European Commission under the Health Programme 2008–2013 [7]. The title of the conference, which saw the participation of international experts and representatives of the European Commission and EU Member States, was “Harmonising European Initiatives on Biocontainment Laboratories” (HEIBL). This article summarizes the consensus reached on a series of critical issues.

## Does Europe Need New BSL-4 Laboratories?

To effectively respond to the threat of a new EID or bioterrorism, a rapid and effective diagnosis is crucial, as is cooperation between existing BSL-4 laboratories. In recent years, the European Network of BSL-4 laboratories has worked to share and harmonize the practices and experience of the laboratories operating in Europe, and the European Union (EU) is committed to further supporting the improvement of early warning, alert, and response to serious cross-border threats to health caused by dangerous pathogens (as set down in Article 168 of the Treaty on the Functioning of the EU). However, the most urgent issue today is how to ensure long-term sustainability of the existing facilities. At present, there are eight recognized (i.e., approved under national regulations) BSL-4 laboratories in the EU (Table 1), plus one in Switzerland and at least four more are in the planning stage or under construction in the Netherlands, Italy, and Germany. All together, the eight facilities in the EU provide a total containment area of approximately 1,800 m^2^ divided into at least 20 subunits and are used for the safe handling of Risk Group-4 *Arenaviridae*, *Bunyaviridae*, *Filoviridae*, and *Paramyxoviridae*.

**Table 1:** European Network of Biosafety-Level-4 Laboratories: Facilities for diagnostics and research operating in the EU.

Country	Organization
France	Laboratoire P4 Jean Merieux, Inserm, Lyon
Germany	Bernhard Nocht Institute for Tropical Medicine, Hamburg
Germany	Philipps Universität Marburg, Marburg
United Kingdom	Health Protection Agency–Centre for Infections (CfI), London
United Kingdom	Centre for Emergency Preparedness and Response, Porton Down
Sweden	Swedish Institute for Communicable Disease Control, Solna, Stockholm
Italy	National Institute for Infectious Diseases IRCCS “L. Spallanzani,” Rome
Hungary	National Center for Epidemiology, Budapest

Building and maintaining high biosafety-level laboratories, with their complex structural design and the need for highly skilled personnel, requires a huge investment. Although private companies or academic institutions could contribute to cover operational costs, BSL-4 laboratories rely mainly on national resources; therefore, planning without the necessary foresight would be similar to creating cathedrals in the desert. Instead, every effort should be made to ensure that all countries have access to BSL-4 diagnostics through European networks such as the newly funded ERINHA (European Research Infrastructure on Highly Pathogenic Agents) [8] and the long-lasting European Network of P4 laboratories, now part of the QUANDHIP project (Quality Assurance Exercises and Networking on the Detection of Highly Infectious Pathogen) [9], and through the implementation of standard operating procedures for rapid transport of samples. On the other hand, the threat of an uncontrolled proliferation of new facilities, as has been reported in the United States (where it was feared that the number of planned BSL-4 laboratories might increase following the 2001 terrorist attacks [10]), is a matter of concern as it would reduce the financial resources available to each laboratory, and more importantly, the cost of the new training needs would make it impossible to meet the growing demand of skills and expertise. In addition, concentrating the clinical samples in few laboratories with proven proficiency in detecting emerging pathogens, and round-the-clock capacity, would be crucial to a comprehensive, rapid, and sensitive differential diagnosis.

## How Can We Improve Biosafety, Biosecurity, and Training for BSL-4 Personnel?

Biosafety and biosecurity are a constant matter of debate in the BSL-4 community. By definition, the term *biosafety* describes the working practices and structural characteristics that prevent unintentional release or exposure to pathogens, while *biosecurity* refers to all measures implemented to prevent unauthorised access to, misuse of, or intentional release of dangerous biological materials.

The purpose of high containment laboratories is to protect the health and safety of the individual and the community, and public health authorities must make sure that rules and guidelines are reviewed and updated. Unfortunately, this is not always done on the basis of documented evidence of effectiveness, causing concern to BSL-4 workers, as testified by the articles published by American and European BSL-4 scientists [11], [12] strongly opposing the “two-persons rule” describing the minimum number of persons required in the laboratory at all times, irrespective of procedures performed. Scientists and biosafety officers should work closely together and agree on biosafety rules drawn from experience, avoiding unnecessary restrictions that would create discomfort. BSL-3 and BSL-4 scientists should demand more attention to biosafety in the basic curriculum of graduates in Medicine and Veterinary Medicine, Biology, Engineering, and Architecture. This would help to create a biosafety culture and improve our capacity to effectively respond to outbreaks of disease caused by known and unknown dangerous pathogens. With regard to biosecurity, all European laboratories have their procedures in place to regulate access, inventory, and safeguarding of samples; strict rules for sample sharing; and security checks on staff. These procedures sometimes vary slightly in different countries, but they share some common aspects: access to the high containment area is strictly controlled and traceable through personal badges and security cameras, samples are stored inside the laboratory, and all aliquots must be accounted for. Samples are shared on written request only between officially recognized BSL-4 laboratories, and therefore between scientists who know each other well (the BSL-4 community is a small one).

Ideally, Level-4 training should be provided in a BSL-4 facility, where safety rules and technical restrictions (e.g., working space, air supply) limit the maximum number of people allowed in the laboratory at the same time. For this reason, it is generally provided on a one-to-one basis to highly committed individuals selected on criteria such as skills in good microbiological practice, physical fitness, and absence of a criminal record; other than this, there is a high variability in the training practices of European BSL-4 laboratories, with most labs requiring also fire practice, first aid, and rescue courses, and an additional assessment of competency for working with animals. Some believe that mock laboratories could be used to acquaint the would-be BSL-4 worker with specific sets of practices and procedures, but we maintain that this approach would not substitute for on-site BSL-4 tutoring of scientists with extensive experience of handling infectious agents. Practical training should take into account specific biosafety requirements, such as those dictated by the type of laboratory (e.g., suit-based or cabinet line). An interdisciplinary approach—that is, training courses where health care workers from different disciplines (physicians, microbiologists, nurses, engineers specialized in construction and maintenance of high containment facilities, and biosafety professionals) can be trained together—would be extremely beneficial, as proven by the experience with the ETIDE project, a 3-year EU-funded training programme that ran from 2006 to 2009 [6]. The development of a basic common training protocol, acceptable to all BSL-4 laboratories, would hopefully allow an easier exchange of trained staff in the future, and different aspects of this issue are being addressed by two other EU-funded projects, ERINHA and QUANDHIP [8], [9].

## Does Europe Need Mobile Laboratories?

Most of the viruses handled in BSL-4 laboratories cause outbreaks in developing tropical and subtropical countries in Asia, South America, and Africa, where mobile laboratories have been used to provide early diagnostic capacity and to support medical, surveillance, and outbreak management teams, thus circumventing the well-known obstacles to sample transport [13]–[18]. Another important advantage of employing mobile facilities in the field is the possibility to gain access to biological materials needed to research and validate new diagnostic assays, as most of them are home-made.

A new EU-funded project that aims to develop deployable laboratories to assist EU and non-EU countries in case of outbreaks caused by human-to-human transmitted pathogens (known or unknown) is now in its first year of activities. The overall objective is to create a collaborative network of European and African institutions that would then operate in common mobile units during outbreaks. Two main units are foreseen to be deployed in sub-Saharan Africa; a third one should be based in Europe for training but may also be deployed in EU neighbouring countries in case of need. These future mobile laboratories should have the capacity to detect pathogens in clinical specimens by molecular assays (mainly real-time PCR) as well as by other methods (ELISA or other antibody-based techniques), as appropriate depending on the outbreak, and a module for detecting unknown pathogens. They may also include basic blood chemistry and haematology to support patient case management, modules for detecting nonviral human pathogens, as well as plant and veterinary agents with high economical or environmental impact, and Class A agents of potential deliberate release. To avoid competition, efforts should be coordinated with other already existing mobile laboratory services such as those of the CDC (U.S. Centers for Disease Control and Prevention) and the Public Health Agency of Canada. Specific training programmes should be designed together with African countries, taking into account factors such as different legislations, policies, public health implications, logistics, and equipment available in each of those countries.

## Summary of Findings and Next Steps

The experience with the existing BSL-4 laboratory networks [2] has been valuable in facilitating information and resource sharing, scientific exchange and training, and organizing external quality control assays; the result of the 2-day conference held in 2010 [7] was a consensus on some key issues that have represented a new starting point for the future of the fight against HIDs:

- **Sustainability**. Limitations in funding together with the risk of proliferation of new facilities in Europe and North America pose the question of their long-term sustainability. Resources need to be identified for the maintenance of their diagnostic capability, training, and research. In particular, funding should be provided to continue the implementation of interlaboratory collaboration, including coordinated quality control assays, training, and professional development.
- **Biosafety and Biosecurity**. A better interaction between biosafety professionals and researchers should be encouraged, as well as the adoption of policies and practices that are evidence-based. A consensus should be found to allow the exchange of valuable samples or reagents between designated high containment facilities, and formal agreements addressing security issues should be established between European countries to allow the exchange of researchers, and to grant access to BSL-4 laboratories to scientists from countries where there are no such facilities.
- **Training**. Future training and Continuous Professional Development courses or workshops should be designed to include all categories of healthcare professionals (physicians, nurses, laboratory workers, first responders) as well as the engineers and biosafety professionals that support the BSL-4 infrastructure.
- **Interoperability and the “One Health” Approach**. As human and animal health are intricately connected, human, veterinary, and military laboratories should work towards a full interoperability according to the “One World–One Health” principle [19]. We need to continue working on standards and common procedures and on a legal framework to serve as terms of reference for Member States; we also need to address the new challenges in the framework of the international legal provisions of the EU and the International Health Regulations of the World Health Organization [20]. It is vital that the EU and national authorities continue to support the existing networks and facilitate links between countries with BSL-4 facilities and other countries in need of assistance.
- **European Laboratory Team for EID Outbreak Response**. It is time to create an international and multidisciplinary team of experts and deployable equipment (mobile laboratories) to assist developing countries in the critical early phases of an outbreak, when rapid diagnosis is essential for successful outbreak management. This means also designing common training programmes that take into account the needs of countries with different legislations, logistics, and infrastructure.

Since the 2010 conference, European BSL-4 laboratories have strengthened their programme of coordinated quality assurance exercises under the QUANDHIP project that started in 2011 [9] and have become part of the larger ERINHA consortium funded under the 7^th^ Framework Programme of the European Commission [8], the goal of which is to facilitate access to the BSL-4 research infrastructure by qualified scientists from EU countries. Efforts to establish a network of European and African institutions for the creation of a mobile laboratory are also underway. The future of the European BSL-4 community is in the global coordination of the fight against HIDs.
